# Editorial: Brain-inspired autonomous driving

**DOI:** 10.3389/fnbot.2025.1543115

**Published:** 2025-01-16

**Authors:** Elishai Ezra Tsur, Gianluca Di Flumeri, Hadar Cohen Duwek

**Affiliations:** ^1^The Neuro-Biomorphic Engineering Lab (NBEL), Department of Mathematics and Computer Science, The Open University of Israel, Ra'anana, Israel; ^2^Department of Molecular Medicine, Sapienza University of Rome, Rome, Italy

**Keywords:** navigation & control, spiking neural network (SNN), control theorem and control engineering, neurophysiologic assessment, adaptive control

Autonomous driving is one of the hallmarks of artificial intelligence. Neuromorphic, brain-inspired computing architectures, are revolutionizing vehicular autonomy through biomimetic approaches (Tsur, 2021). They can dramatically impact the multidimensional landscape of autonomous driving, extending across critical domains such as control (DeWolf, 2021), navigation (Novo et al., 2024), and neurophysiological assessment (Wilson and Russell, 2003). Particularly, these brain-inspired computational systems leverage spiking neural networks (SNNs) and event-driven processing to enhance real-time perception (Cohen Duwek and Tsur, 2021), decision-making (Shalumov et al., 2021), and adaptive learning capabilities (Ehrlich et al., 2022). This article collection revolves around the state of the art of neuromorphic systems, designed to support autonomous driving across those dimensions.

Neuromorphic control is posed to significantly contribute to autonomous behavior by leveraging spiking neural networks-based energy-efficient computational frameworks. In this collection, Halaly et al. explored neuromorphic implementations of four prominent controllers for autonomous driving: pure-pursuit, Stanley, PID (Proportional – Integral – Derivative), and MPC (Model Predictive Control), using CARLA (Gomez-Huelamo et al., 2021), a physics-aware simulation framework. Their results show that those neuromorphic control models converge to their optimal performances with merely 100–1,000 neurons. They also highlight the importance of hybrid conventional and neuromorphic designs, as was suggested here with the MPC controller. This MPC was later extended to support adaptive behavior and to address unforeseen situations such as malfunctioning and swift steering scenarios (Halaly and Tsur, 2024). Further in this Research Topic, Lian et al. addressed the importance of adaptive control for changing road conditions by proposing a neural model for deriving road adhesion coefficient (the maximum friction coefficient between tire and road surface) and tire cornering stiffness (affected by road friction and tire slip angle). Those parameters can be used to improve the design of the vehicle's dynamic model and enhance the controller's robustness.

Autonomous driving systems (ADSs) often comprise multimodal sensing, including cameras, LiDARs, IMUs, and GPS, for real-time object detection, semantic segmentation, planning, and control (Zhao et al., 2024). LiDAR-driven neural perception is an important stepping stone toward the design of self-navigating vehicles (Shalumov et al., 2021). In this Research Topic, Lee and Jeong propose a neural reinforcement model, they termed the “Velocity Range-based Evaluation Method” in which LiDAR data is used to provide path planning in a map-less environment.

Finally, in the evolutionary scenario of autonomous driving technologies, the transitional phase characterized by partial vehicle autonomy presents critical challenges in human-machine interaction (Xing et al., 2021). The emerging paradigm necessitates a sophisticated, bidirectional monitoring system that transcends traditional human-vehicle interfaces. In fact, the vehicle must develop robust capabilities for comprehensive driver state monitoring, including advanced sensor fusion techniques for enabling holistic driver condition evaluation, to determine whether the driver is able to intervene and take control (Ansari et al., 2022). Empirical research suggests that effective human-autonomous system collaboration requires not just technological sophistication, but a deep understanding of human cognitive and physiological variability (Unni et al., 2022). The ultimate goal is to develop a symbiotic human-machine interface where autonomous systems act as collaborative partners rather than mere technological substitutes, enhancing overall transportation safety and efficiency. In this regard, in this article collection, Giorgi et al. propose an integrated framework to assess the driver's mental fatigue in real time using electroencephalographic, electrooculographic, photoplethysmographic, and electrodermal activity. Their holistic approach showed that the most sensitive and timely parameters are those related to brain activity. To a lesser extent, those related to ocular parameters are also sensitive to the onset of mental fatigue but with a delayed effect.

In conclusion, the confluence of neuromorphic computing, advanced sensing technologies, and sophisticated human-machine interaction represents a pivotal transformation in autonomous driving. This Research Topic illuminates the multifaceted challenges and innovative solutions emerging at the intersection of artificial intelligence, neuroscience, and automotive engineering. From energy-efficient neural controllers to adaptive perception systems and comprehensive driver state monitoring, the research demonstrates that autonomous driving is far more than a technological challenge—it is a complex socio-technical ecosystem.

The future of autonomous vehicles might lie not in complete human replacement, but in creating intelligent, responsive systems that collaborate seamlessly with human operators. By integrating biomimetic computational approaches, advanced sensor fusion, and a nuanced understanding of human cognitive variability, we move closer to a transportation paradigm that prioritizes safety, efficiency, and harmonious human-machine interaction.
